# Pain reduction through combined spinal cord stimulation and exercise therapy after spinal extradural arachnoid cystectomy: a case report

**DOI:** 10.1186/s40981-025-00811-x

**Published:** 2025-10-16

**Authors:** Akari Ikemura, Daigo Shiroki, Satoshi Ohga, Takafumi Hattori, Yoko Sugiyama, Yuko Kito, Maki Mizogami, Takako Matsubara, Hiroki Iida

**Affiliations:** 1 Anesthesiology and Pain Relief Center, Central Japan International Medical Center, 1–1 Kenkounomachi, Minokamo, Gifu 505–8510 Japan; 2 Department of Rehabilitation, Central Japan International Medical Center, 1–1 Kenkounomachi, Minokamo, Gifu 505–8510 Japan; 3 Faculty of Rehabilitation, Kobe Gakuin University Graduate School, 518 Arise, Ikawadani-cho, Nishi-ku, Kobe, Hyogo 651–2180 Japan

**Keywords:** Spinal extradural arachnoid cysts, Spinal cord stimulation, Exercise therapy

## Abstract

**Background:**

Spinal extradural arachnoid cysts (SEACs) can cause persistent pain after surgery. Combining spinal cord stimulation (SCS) with structured exercise therapy may aid long-term pain modulation.

**Case presentation:**

A teenage female with over two years of severe rib pain and bilateral leg pain/numbness was diagnosed with SEACs and underwent resection. A trial of SCS alleviated leg symptoms; rib pain persisted. After SCS implantation, leg symptoms resolved, and a gradual exercise progressed from pain-free stretching to low-intensity lower-limb exercises. Over 12 months, rib pain decreased and skeletal muscle mass increased. Pressure pain threshold and conditioned pain modulation improved, suggesting reduced sensitization and enhanced descending inhibition.

**Conclusions:**

This case suggests that SCS may provide early pain relief, enabling initiation of structured exercise, associated with sustained pain reduction, improved pain modulation, and functional recovery. These observations suggest the SCS combined with exercise may be a useful option for selected patients with movement-limiting multifocal pain.

## Background

Spinal extradural arachnoid cysts (SEACs) are rare spinal lesions that account for approximately 1% to 3% of spinal tumors. They predominantly affect males in their 20s, 30s, or 50s and are most commonly located in the thoracic spine, although they can occur at any spinal level [1]. Clinically, patients with SEACs may present with back pain, progressive sensory disturbances, pain radiating to the flanks or lower-extremities, or symptoms of spinal cord compression. Persistent pain is frequently observed even after surgical intervention [2, 3]. While conservative follow-up is typically recommended in mild cases, additional surgical intervention is required when symptoms worsen.

Multimodal approaches have recently gained attention for chronic pain management. This strategy aims to overcome the limitations of single-modality treatment by integrating interventional procedures, pharmacological therapies, and exercise. Spinal cord stimulation (SCS) is a minimally invasive intervention that delivers rapid analgesia via electrical stimulation of the posterior spinal cord [4]. SCS is particularly effective for neuropathic and ischemic pain but is less effective for nociceptive pain conditions.

Exercise therapy is a noninvasive treatment widely recommended for chronic pain conditions, and it promotes analgesia via exercise-induced hypoalgesia, modulation of pain sensitization, and enhancement of descending pain inhibitory pathways. However, initiating exercise can be challenging for patients experiencing severe pain.

We report a case in which SCS was combined with structured exercise therapy to explore its potential for facilitating exercise initiation and enhancing long-term pain modulation in a patient with movement-limiting pain. To our knowledge, reports describing the combined use of SCS and structured exercise therapy for refractory pain following SEACs resection are scarce, and this case may provide useful insights for multidisciplinary pain management in such complex conditions.

## Case presentation

Written informed consent was obtained from the patient for the publication of this case report.

**Fig. 1:**
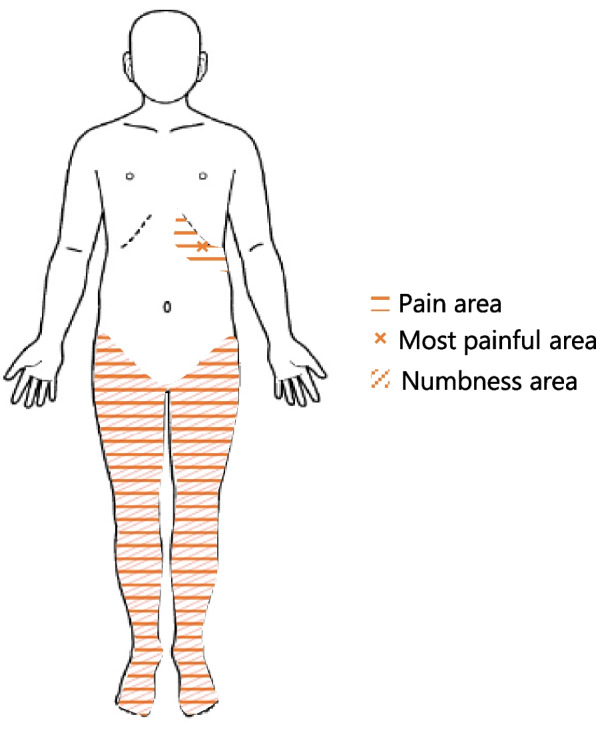
Pain and numbness locations

A teenage female high school student presented with a more than two-year history of severe pain at the inferior border of the left costal margin (approximately the 10th rib), along with bilateral lower-limb pain and numbness (Fig. 1). Magnetic resonance imaging revealed SEACs extending from T7 to T11, and the patient underwent cystectomy two years after symptom onset.

However, six months after surgery, the patient experienced a relapse of severe pain localized to the left 10th rib area and bilateral leg pain and numbness. These symptoms were initially managed with mirogabalin (30 mg/day) and various nerve blocks, including thoracic radiofrequency medial branch denervation (i.e., thermal coagulation of the medial branch nerves innervating the thoracic facet joints), but these interventions yielded no meaningful improvement.

Seven months post-surgery, a trial of SCS at the T8–T12 level provided marked relief of the patient’s leg symptoms. The numerical rating scale (NRS) score for leg pain decreased from 5 to 0, and the numbness also resolved, allowing the discontinuation of mirogabalin. Based on the positive response, permanent SCS implantation (Proclaim™ XR 7, Abbott) was performed, with the lead placed epidurally at T8–T9 and stimulation delivered in BurstDR (Continuous mode). However, the left rib pain remained unchanged. Unlike the leg symptoms, the rib pain was not accompanied by numbness or other sensory disturbances and showed no response to SCS.

Twenty days before implantation, the patient was referred to a chronic pain rehabilitation program aimed at improving her functional capacity and pain-coping strategies [5]. At the initial assessment (day 0), she reported persistent left rib pain (NRS 10) and residual leg pain (NRS 5). Quantitative sensory testing (QST) revealed a markedly reduced pressure pain threshold (PPT) over the left rib (7.7 N), indicating localized hyperalgesia [6]. Conversely, the PPT measured over the tibialis anterior muscle was within normative ranges for healthy females [7], indicating the absence of widespread sensitization. Conditioned pain modulation (CPM), assessed via a standardized earlobe pressure pain stimulus, was within normal limits (37.8%) [8]. Pain-related disability, assessed using the pain disability assessment scale (PDAS) [9], was severe (score of 21), restricting daily activities and preventing school attendance. Initially, rehabilitation consisted exclusively of gentle, pain-free stretching exercises, along with pain education to promote safe movement strategies. On day 20, an implantable pulse generator was successfully placed. After implantation, both leg pain and numbness resolved completely, while the rib pain remained severe (NRS 8). Consequently, low-intensity aerobic lower-limb exercises (walking and pedaling) were carefully introduced at a dose of 10 min/day, five days per week, to facilitate systemic analgesia. The patient’s adherence to the exercise program was assessed through self-report.

At three months post-treatment, PDAS improved from a baseline of 21 to 0, indicating resolution of pain-related disability. However, rib pain at rest persisted (NRS 7), following the resumption of school attendance and prolonged sitting time. The increase in daily pain exposure may have contributed to sustained peripheral sensitization and descending pain inhibition dysfunction, as reflected by persistently low PPT and a marked decrease in CPM from 37.8% to 6.5%. In response, exercise duration and intensity were progressively increased with careful monitoring to avoid exercise-induced hyperalgesia. To better evaluate intervention effects, absolute skeletal muscle mass (SMM) of the lower-limb and trunk was measured using bioelectrical impedance analysis (InBody S10, Tokyo, Japan). To account for physical growth, relative SMM values were calculated: SMM adjusted by weight (%SMM) = SMM (kg)/weight (kg) × 100 [10]. At this point, lower-limb SMM was 13,500 g (%SMM: 15.9%) and trunk SMM was 21,100 g (%SMM: 24.8%).

At five months post-treatment, rib pain fluctuated, particularly during prolonged inactivity (e.g., school vacations or return to classes). At rest, rib pain remained moderate (NRS 5) but improved with physical activity. PPT improved from 7.7 N to 10.0 N, and CPM recovered from 6.5% to 28.2%, reflecting reduced hyperalgesia and restoration of endogenous pain inhibition. Lower-limb SMM increased by 900 g, reaching 14,400 g (%SMM: 17.4%).

**Fig. 2:**
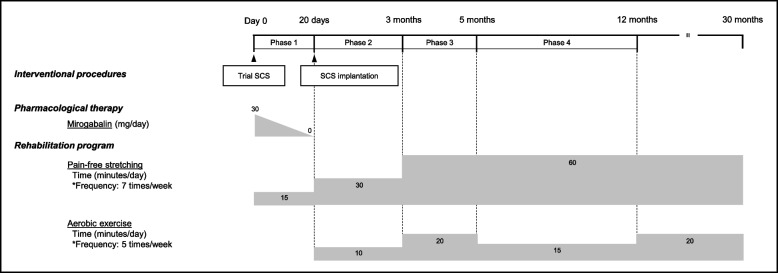
Time course of treatment and follow-up over 30 months. The diagram illustrates the sequence of interventions and clinical progression, including a trial of spinal cord stimulation (SCS), permanent SCS implantation, pharmacological therapy, and the subsequent rehabilitation period. Daily doses of mirogabalin (mg/day) and exercise load (min/day) are shown. Phase 1: Only pain-free stretching exercises were performed due to severe rib pain. Phase 2: Low-intensity aerobic lower-limb exercises were added as leg pain decreased after the SCS device implantation. Phase 3: The exercise load was progressively increased in response to persistent peripheral sensitization and diminished descending pain inhibition. Phase 4: The exercise load was maintained following reduced hyperalgesia and restoration of endogenous pain inhibition. Abbreviation: SCS, spinal cord stimulation

**Fig. 3:**
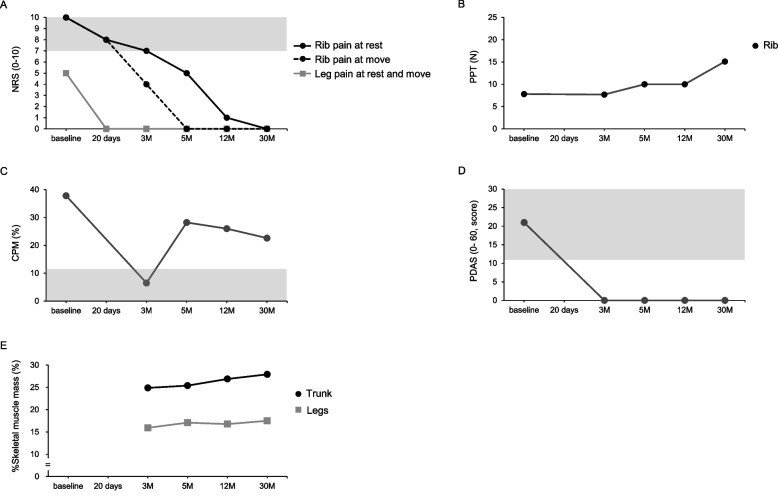
Changes in clinical measures from initial treatment to 30 months of follow-up. The figure illustrates the time course of changes in (A) rib pain and leg pain and numbness intensity (NRS), (B) PPT of the left rib, (C) CPM, (D) pain-related disability (PDAS), and (E) %SMM of the lower-limbs and trunk. The gray shading indicates (A) severe pain, (C) diminished descending pain inhibition, and (D) pain-related disability. Abbreviation: NRS, numerical rating scale; PPT, pressure pain threshold; CPM, conditioned pain modulation; PDAS, pain disability assessment scale; SMM, skeletal muscle mass

After one year of continued exercise, rib pain at rest improved to NRS 0–1, and CPM remained within normal limits. Lower-limb SMM was maintained, and trunk SMM increased substantially by 2,000 g, from 21,100 g (%SMM: 24.8%) to 23,100 g (%SMM: 26.8%). The patient successfully graduated from high school, started college, and continued self-managed care without requiring outpatient follow-up. Consequently, removal of the SCS device was scheduled for the following summer vacation as part of the long-term management strategy, given the patient’s stable condition and absence of pain recurrence. A summary of the clinical course and outcomes is presented in Fig. 2 and 3.

## Discussion

This case report highlights the synergistic benefits of combining SCS with structured exercise therapy and demonstrates its effectiveness in achieving sustained pain relief, improving pain modulation, and functional recovery. Although SCS is widely recognized as an effective intervention for neuropathic pain, its role in facilitating active rehabilitation is poorly documented. In the current case, the immediate analgesic effect of SCS enabled early initiation and sustained engagement in exercise therapy, which supported long-term pain management and functional improvement.

An important observation was the marked improvement in CPM from 6.5% to 28.2% after prolonged exercise. Given that impaired CPM is characteristic of many chronic pain conditions, this change suggests the enhancement of descending pain inhibitory pathways, potentially mediated by exercise-induced neuroplasticity. A previous study indicated improvements in CPM after approximately 12 weeks of low- to moderate-intensity exercise [11], consistent with our observations. In parallel, PPT improved from 7.7 N to 10.0 N, indicating reduced mechanical hyperalgesia and gradual desensitization of peripheral nociceptors through progressive, non-painful physical activity. This finding aligns with existing evidence that graded exercise can downregulate nociceptive afferent activity and reduce peripheral sensitization [12, 13]. A critical factor contributing to the success in this case was the early analgesia provided by the SCS, which interrupted the cycle of inactivity, fear avoidance, and deconditioning commonly observed in patients with chronic pain. SCS effectively bridged the transition to active rehabilitation by reducing pain intensity to allow safe and sustained physical activity.

Improvement in pain outcomes was accompanied by an increase in %SMM of 1.5% for the lower-limbs and 2.0% for the trunk. These changes are clinically meaningful because skeletal muscle contributes to pain modulation through biomechanical support and the release of anti-inflammatory myokines, such as interleukin-6, which influence central pain processing [14, 15]. These neuromuscular adaptations suggest that exercise not only produces immediate hypoalgesic effects but also contributes to long-term pain control through structural and systemic mechanisms. The potential risk of exercise-induced hyperalgesia was avoided through a carefully individualized rehabilitation approach that emphasized gradual progression and patient tolerance. This strategy prevents symptom exacerbation and promotes adherence, underscoring the importance of personalized exercise prescriptions for chronic pain management.

Although limited by its single-case nature, this report highlights the potential clinical value of combining SCS with structured exercise therapy as part of a multimodal pain management approach. Future research should aim to define optimal exercise parameters and elucidate the neurophysiological mechanisms underlying the analgesic effects of these combined interventions.

In conclusion, this case suggests that combining SCS with structured exercise therapy may improve pain intensity, descending pain inhibition, peripheral sensitization, and physical function. These findings may support the potential effectiveness of combining interventional therapy with exercise therapy, particularly for patients with multiple severe pain events that initially preclude exercise participation.

## Data Availability

The datasets used and/or analyzed during the current study are available from the corresponding author on reasonable request.

## Abbreviations

CPM: Conditioned pain modulation
NRS: Numerical rating scale
PDAS: Pain disability assessment scale
PPT: Pressure pain threshold
QST: Quantitative sensory testing
SCS: Spinal cord stimulation
SEACs: Spinal extradural arachnoid cysts
SMM: Skeletal muscle mass
TA: Tibialis anterior
